# Profiling of serum metabolites in advanced colon cancer using liquid chromatography-mass spectrometry

**DOI:** 10.3892/ol.2020.11510

**Published:** 2020-04-03

**Authors:** Yang Zhang, Yechao Du, Zheyu Song, Suoning Liu, Wei Li, Daguang Wang, Jian Suo

**Affiliations:** The First Hospital of Jilin University, Changchun, Jilin 130021, P.R. China

**Keywords:** colon cancer, lymph node metastasis, serum metabolites, liquid chromatography-mass spectrometry, prognosis

## Abstract

Lymph node metastasis remains a key factor that affects the prognosis of patients with colon cancer. The aim of the present study was to identify and evaluate serum metabolites as biomarkers for the detection of tumor lymph node metastasis and the prediction of patient survival. The present study analyzed the metabolites in the serum of patients with advanced colon cancer both with and without lymph node metastasis. Blood samples from 104 patients with stage T3 colon cancer were collected and analyzed using liquid chromatography-mass spectrometry. The metabolites were structurally confirmed with data from the Human Metabolome Database. The association between the serum metabolites and the clinicopathological characteristics and survival time of patients from the present study was analyzed. Overall, 227 different metabolites were identified in the serum of patients with stage T3 colon cancer with or without lymph node metastasis. Furthermore, 17 of these metabolites may potentially distinguish those patients with lymph node metastasis from those patients without. In addition, five factors, including abscisic acid, calcitroic acid and glucosylsphingosine presence in the serum, age and sex, were identified as independent predictors for lymph node metastasis (P<0.05). Furthermore, three factors, including abscisic acid, calcitroic acid and glucosylsphingosine presence in the serum were independent predictors for patient survival (P<0.05). In conclusion, the serum levels of abscisic acid, calcitroic-acid and glucosylsphingosine may be considered as potential biomarkers to predict the occurrence of lymph node metastasis and the survival time of patients with colon cancer.

## Introduction

Colon cancer has the second highest morbidity and mortality rates in the United States with 102,000 new cases and 51,000 deaths (1,2), accounting for >1,000,000 newly diagnosed cases and up to 500,000 cancer-associated mortality cases estimated per year between 2016 and 2019 worldwide (3). Patients with early stage disease may present with no specific symptoms or clinical manifestations; however, once symptoms occur, the disease may have already progressed to an advanced stage (4). This delays the opportunity to provide the patient with curative surgery, and thereby increases the risk of mortality (5). Early detection methods for colon cancer include the fecal occult blood test, the analysis of certain gastrointestinal tumor markers, such as CEA and CA19-9, in the serum and the colonoscopy (6–11). Fiber colonoscopy is the best screening method for the early diagnosis of colon cancer, compared with the fecal occult blood test and analysis of serum biomarkers, which are not very reliable due to their low sensitivity and specificity; however, fiber colonoscopy is an invasive and expensive procedure (9). Tumor metastasis, including lymph node metastasis, is the first cause of mortality in these patients (6–11). Further identification and evaluation of serum biomarkers could therefore help clinicians to detect colon cancer early or predict the prognosis and treatment responses in order to successfully treat patients.

Currently, there are numerous approaches to detect biological markers or metabolites in human fluid samples, including serum, sputum, bile and aqueous humor for tumor diagnosis. For example, the detection of gene mutations in serum samples could specifically and sensitively differentiate the disease stages (12–17), whereas analysis of serum metabolites could help diagnose colon cancer early or predict disease progression (18–22). Detection and evaluation of serum metabolomics have been used to better understand the pathogenesis and progression of colon cancer (23). Further studies could identify novel biomarkers that may be used as diagnostic tools in patients with colon cancer. To date, the popularity of modern chromatography, mass spectrometry and other detection techniques, including nuclear magnetic resonance (NMR), could help the discovery of numerous biological metabolites, such as xanthine, hypoxanthine, succinate, N2, N2-dimethylguanosine, adenine, citraconic acid, 1-methylguanosine and d-mannose, some of which were proven to be specific and sensitive biomarkers in cancer research (18–22). Previous studies analyzing serum metabolomics in patients with colorectal cancer or colorectal polyps compared with healthy subjects by using liquid chromatography-mass spectrometry (LC-MS), NMR or mass spectrometry (MS) reported different metabolites, including xanthine, hypoxanthine and d-mannose, as potential biomarkers (18,19). However, to the best of our knowledge, only a few studies compared the serum metabolomics of patients with stage Tumor (T) 3 colon cancer with or without lymph node metastasis (24). In the present study, patients with T3 colon cancer were selected to assess their serum metabolites in order to analyze patients with colon cancer at an earlier disease stage, detect lymph node metastasis and predict their prognosis.

## Materials and methods

### 

#### Study population

In the present study, 104 patients with stage T3 colon cancer treated at The Department of Gastric, Colonic, Rectal and Anal Surgery, The First Hospital of Jilin University (Changchun, China) between August 2008 and August 2012 were selected. There were 58 males and 46 females with an age range of 49–74 years. Patients underwent tumor resection and were histologically diagnosed with colon cancer according to the American Joint Committee on Cancer Staging Manual (25). Among them, 52 patients were diagnosed with T3 Node (N)0 Metastasis (M)0 and the remaining 52 patients were diagnosed with T3NxM0 (N=1-3). Inclusion criteria were as follows: The tumor was initially diagnosed as colon cancer by endoscopy; and no history of tuberculosis, hepatitis, diabetes and mental illness. The exclusion criteria were as follows: Patients with abnormal data from routine blood, urine, liver and kidney function analysis for whom other diseases could not be ruled out; patients with distant tumor metastasis or other tumors detected by CT or MRI imaging; and female patients who were pregnant, breast-feeding, or for whom the possibility of pregnancy could not be ruled out. These exclusion criteria resulted in 104 patients who were suitable for the present study. The study protocol was approved by the Ethics Committee of The First Hospital of Jilin University (Changchun, China) and all participants provided written informed consent prior to enrolment. The present study was in strict accordance with the 1964 Helsinki Declaration and its later amendments.

#### Blood sample processing and liquid chromatography-mass spectrometry

Fasting blood samples (1.5 ml) were collected prior to surgery, then centrifuged at 1,294 × g and the supernatant was removed, leaving the serum which was stored at −80°C. Subsequently, 150 µl of serum from each participant was thawed at room temperature and added to 500 µl acetonitrile (Merck KGaA) to denature the sample at room temperature. Samples were centrifuged at 10,878 × g for 5 min at −4°C, and supernatants were collected and added to the Eclipse Plus C18 column (2.l × l50.0 mm, 3.5 µm; Agilent Technologies, Inc.). High performance liquid chromatography was performed with the Agilent 1200 system combined with a 6520 accurate electrospray ionization/quadrupole-time-of-flight mass system (Agilent Technologies, Inc.). The gradient program consisted of mobile phase A (0.1% formic acid solution) and mobile phase B (99.9% acetonitrile) with a 0.8 ml/min flow rate, 45°C column temperature and 20 µl injection volume. The gradient program was started from 25% A for 0.0–1.5 min, linearly increased from 25 to 90% A for 1.5–7.0 min, kept at 90% A for 7.0–9.9 min, linearly decreased from 90 to 25% A for 9.9–10.0 min, and equilibrated for 25% A for 10.0–11.0 min. The mass spectrometer was set to a desolvation temperature of 350°C, source temperature of 100°C, capillary voltage of 3.2 kv, mass range between 50 and 1,000, scan time of 1 sec, inter-scan delay of 0.02 sec, desolvation gas (nitrogen) flow rate of 650 l/h, cone voltage of 35 volts, and cone gas (nitrogen) flow rate of 50 l/h. The LC-MS data were subsequently analyzed.

#### Data processing and statistical analysis

The raw mass spectra data collected with the software analyst TF 1.5.1 (AB Sciex) were imported into the Marker View 1.2 (AB Sciex) for processing and included the retention time, peak area and m/z ratio. The Human Metabolome Database (HMDB; www.hmdb.ca) was used to structurally confirm the serum metabolites by comparing the m/z ratio and ion mode between the results from this study and the HMDB. Principal component analysis (PCA) was then applied to the data to distinguish the similarities or differences in the scatterplots of patients with different lymph node development stages. The potential biological variables with statistically significant differences for patients with T3 colon cancer with different lymph node development stages were selected using the two-sample t-test. The data are expressed as the mean ± standard deviation and statistically analyzed using the two-sample t-test. Furthermore, hierarchical clustering analysis was conducted using BRB-Array Tools version 3.6 software (developed by Dr Richard Simo & BRB-Array Tools Development Team) to distinguish patients with stage T3 colon cancer with and without lymph node metastasis. The global median subtraction method (26,27) was used to normalize the intensity of the background interference. In addition, the clinicopathological characteristics of the patients were collected, including sex, age, tumor size, presence of invasion to the blood or lymph vessels, p53 expression, Ki-67 serum level, history of alcohol consumption, tobacco smoking and family tumor history, analyzed using a χ^2^ test or Fisher's exact test. Then independent factors were used to calculate the risk scores for lymph node metastasis as follows: Risk score = probability × 100; probability = eZ/(eZ +1), where e indicates the natural logarithm and Z denotes the value of the logistic regression; Z = B0 + B1X1 + B2X2 + B3X3 + … + BpXp, where B0 denotes the regression coefficient of the constant for the logistic regression, X1 … where Xp denotes the individual variable in the logistic regression, and B1 … where Bp denote the corresponding regression coefficients and P denotes the number of vairables (26,27). The survival data were also collected and analyzed using the Kaplan-Meier curves, the log rank test, multivariate Cox regression and receiver operating characteristic (ROC) curves. All statistical analyses were conducted using SPSS 22.0 (IBM Corp.) and P<0.05 was considered to indicate a statistically significant difference.

## Results

### 

#### Differential profiling of serum metabolites between patients with T3N0M0 and T3N1-3M0 colon cancer

**Figure 1.**
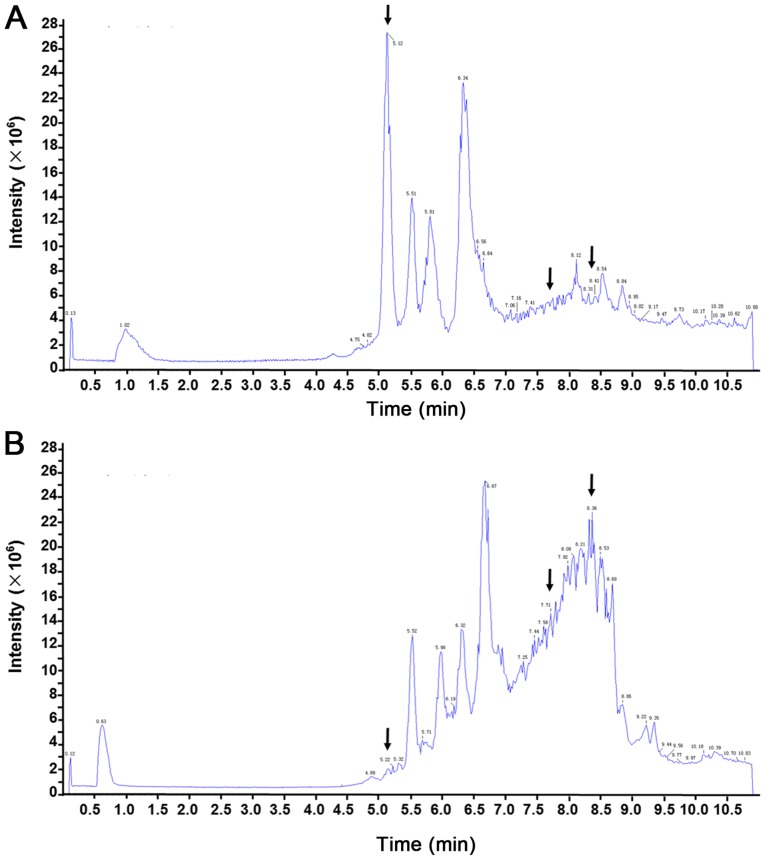
Representative serum total ion current mass spectra for the serum samples from patients with stage T3 colon cancer and different lymph node status. (A) Serum sample from a patient with T3N0 colon cancer. (B) Serum sample from a patient with T3N1-3 colon cancer.

**Figure 2.**
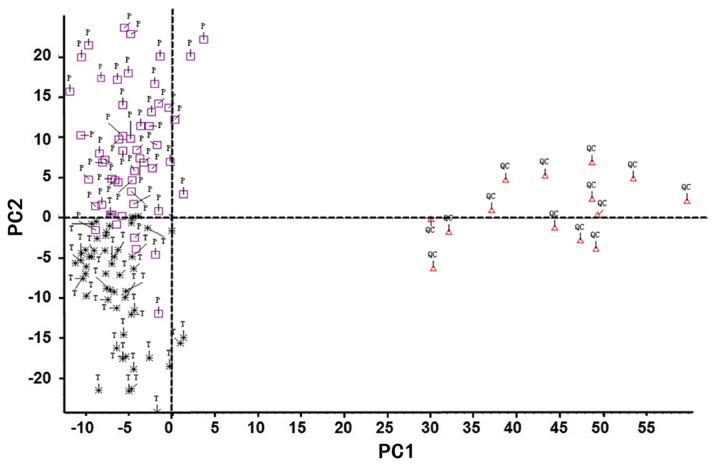
Principal component analysis (PCA) scores and plots of serum metabolites from patients with colon cancer and different lymph node status. Pink square, T3N0 patients. Black star, T3N1-3M0 patients. Red triangle, QC samples. QC, quality control; PC, principal component.

**Figure 3.**
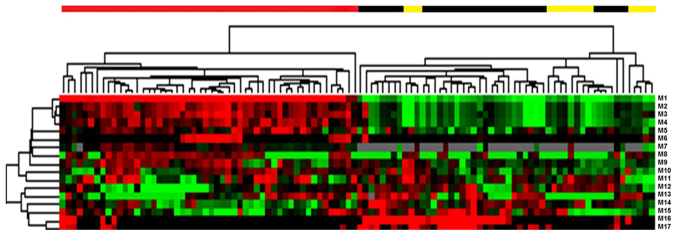
Hierarchical clustering analysis of 17 serum metabolites from patients with colon cancer and different lymph node status. The different colors in the heat map indicate different lymph node states: Red, no lymph node metastasis; Black, T3N1; Yellow, T3N2. M1-M17 correspond to the 17 metabolites presented in Table I.

In the present study, LC-MS was performed to assess the differential serum metabolite profile between blood samples from patients with T3N0M0 and T3N1-3M0 colon cancer. Differences in the total ion current spectra in the serum samples from patients with stage T3 colon cancer and different lymph node development stages were observed in the 11-min retention time, suggesting that lymph node metastasis may induce important metabolic changes in these patients (Fig. 1). A total of 227 metabolites were detected in the serum of patients with T3 colon cancer at different lymph node developmental stages and the differences between the two groups were statistically significant (data not shown; P<0.05). These 227 metabolites were structurally confirmed by comparison with the data acquired from HMDB. PCA, which is a statistical method used to cluster the detected serum metabolites into a smaller number of principal components (PCs), was performed to determine specific metabolic differences among patients. The outliers or discretization trends in samples from patients were distinguished. Subsequently, almost all samples were separated into 2 groups in the PCA plots, suggesting that these serum metabolites may allow the classification of patients according to their stage (Fig. 2). Furthermore, data demonstrated that a total of 17 serum metabolites could potentially distinguish the patients with lymph node metastasis from patients without following two-sample t-test analysis (Fig. 3; Table I).

#### Association between differential serum metabolites and lymph node metastasis

**Figure 4.**
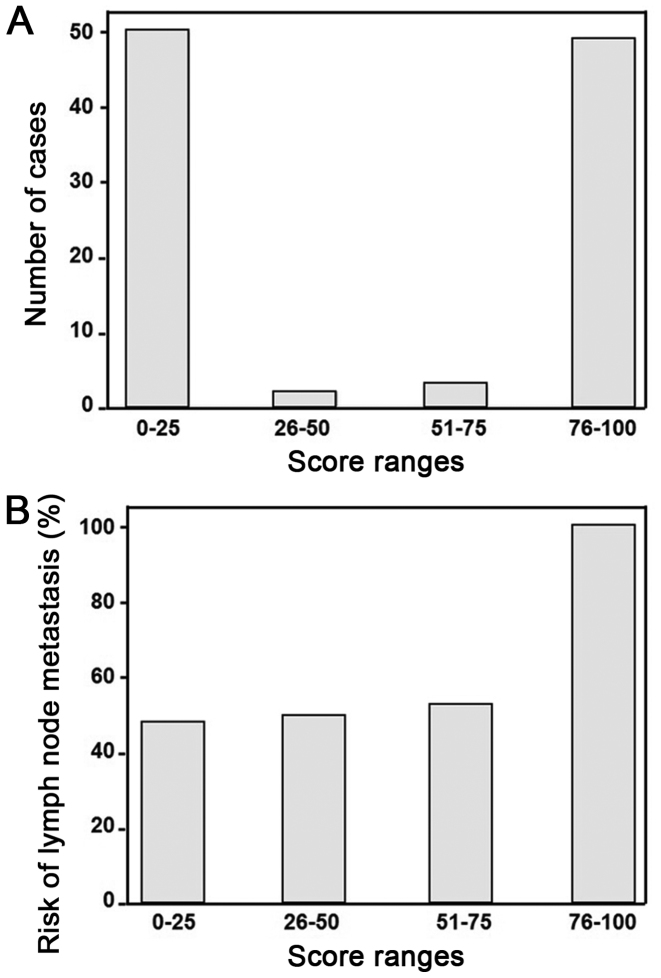
Risk scores for lymph node metastasis in the 104 patients with stage T3 colon cancer. (A) Majority of the risk scores are distributed on both ends of the selected range with only a few distributed in the middle. (B) Risk of lymph node metastasis is associated with the risk score, (the higher the risk score, the higher the likelihood of lymph node metastasis to appear).

**Figure 5.**
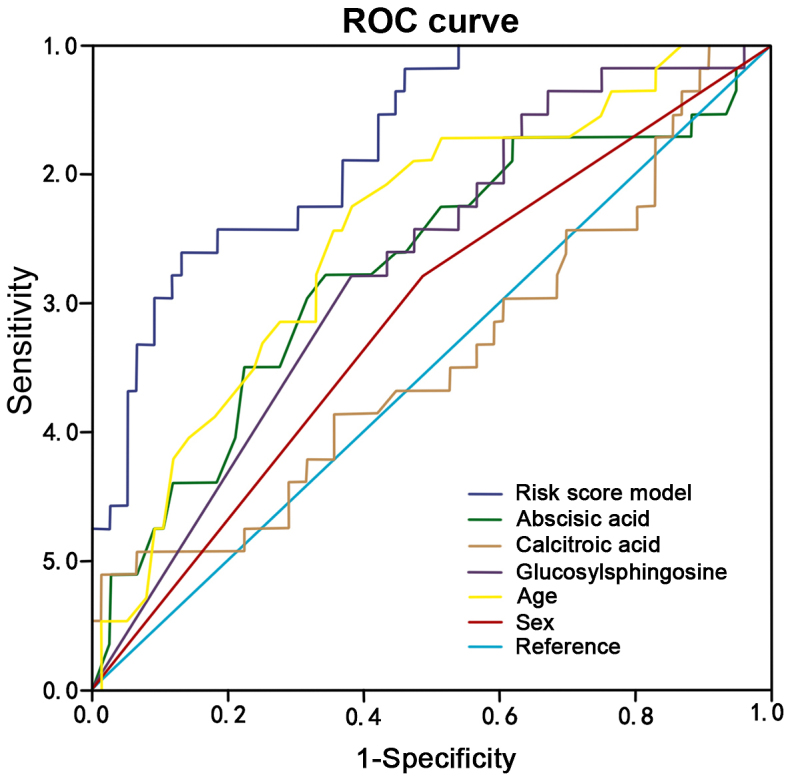
ROC curves of different risk factor. Three serum metabolites and clinicopathological characteristics of patients (sex and age) can predict lymph node metastasis for patients with stage T3 colon cancer. ROC, receiver operating characteristic.

A total of 10 different metabolites from 17 metabolites were selected using the support vector machine method. These data included metabolites, age, sex, tumor size, vascular infiltration, P53, ki-67, history of alcohol consumption, smoking history and family tumor history. These were then analyzed by single-factor logistic regression (Table II). Five independent risk factors, including presence of abscisic acid, calcitroic acid and glucosylsphingosine in the serum, age and sex, were associated with different status of lymph node metastasis in colon cancer were observed. Following multivariate logistic regression analysis, five independent risk factors, including presence of abscisic acid, calcitroic acid and glucosylsphingosine in the serum, age and sex were identified as associated with lymph node metastasis (P<0.05; Table III). These five independent factors were used to calculate the risk scores for lymph node metastasis. The results demonstrated that Z values = −6.403 - 0.44 (abscisic acid) + 0.018 (calcitroic acid) - 0.012 (glucosylsphingosine) + 0.090 (age) −1.141 (sex). Each patient in the present study was assigned a risk score using this formula (Fig. 4). In addition, the results from ROC curves confirmed that the presence of abscisic acid, calcitroic acid and glucosylsphingosine in the serum, age and sex, could be considered as factors for lymph node metastasis in these patients (AUC of abscisic acid, calcitroic acid, glucosylsphingosine, age and sex were 0.844, 0.655, 0.517, 0.655, 0.708 and 0.578, respectively; Fig. 5).

#### Association between differential serum metabolites and patient survival

**Figure 6.**
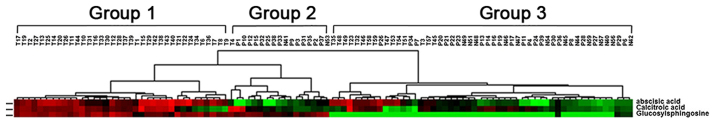
Hierarchical clustering analysis of the serum metabolites abscisic acid, calcitroic acid, and glucosylsphingosine. These three serum metabolites could predict the survival of 104 patients with colon cancer. Group 1, high level; Group 2, intermediate level; Group 3, low level. Higher levels predict better prognosis. Red, green and black are the expression values of metabolites, with high red, low green and medium black.

**Figure 7.**
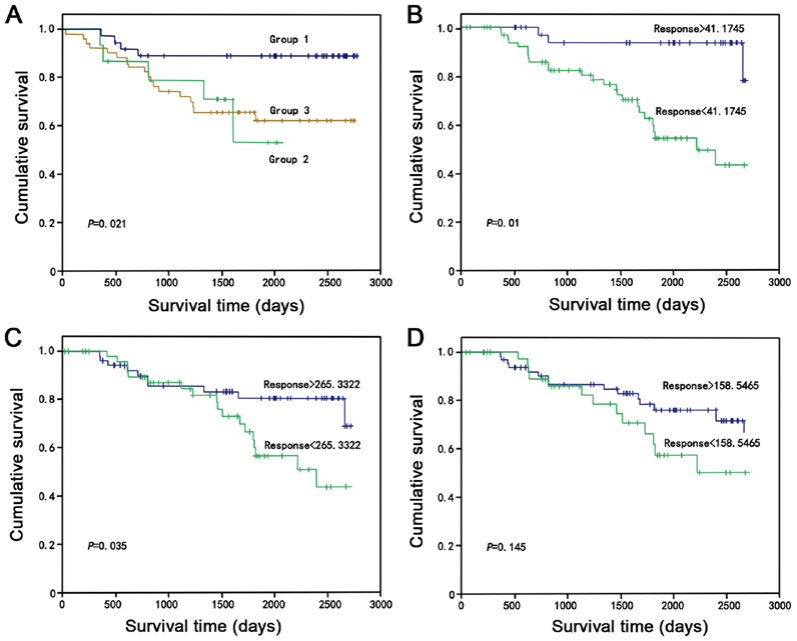
Kaplan-Meier curves predicting the survival of the 104 patients with colon cancer stratified by the three metabolites abscisic acid, calcitroic acid and glucosylsphingosine. (A) Kaplan-Meier curve stratified by the level of all three serum metabolites. (B) Kaplan-Meier curve stratified by the level of abscisic acid. (C) Kaplan-Meier curve stratified by the level of calcitroic acid. (D) Kaplan-Meier curve stratified by the level of glucosylsphingosine.

The association between differential serum metabolites and patient survival was determined. The results demonstrated that the three serum metabolites abscisic acid, calcitroic acid and glucosylsphingosine were independent risk predictors for patient survival (Fig. 6; P<0.05). The hierarchical clustering analysis data are presented in Fig. 6. The results from the Kaplan-Meier curves demonstrated that survival was significantly improved for patients with high levels of these three metabolites compared with patients with medium and low levels (P=0.021; Fig. 7A). The results were also consistent when only one serum metabolite was selected (Fig. 7B, abscisic acid, P=0.01; Fig. 7C, calcitroic acid, P=0.035; Fig. 7D, glucosylsphingosine, P=0.145), and abscisic acid, calcitroic acid and glucosylsphingosine may be considered as potential prognostic markers for all patients with colon cancer (χ^2^=7.725; P=0.021).

## Discussion

The incidence of colon cancer has increased each year over the last decades, and this disease, which markedly alters the quality of life of patients, is associated with a high mortality rate (1,2). Patients with advanced stages of colon cancer cannot receive surgery and are also resistant to chemoradiation therapy; however, recent targeting or immune therapy may serve to control the progression of colon cancer in certain patients (6–11). The present study therefore aimed to establish the profile of serum metabolites of patients with colon cancer, in order to identify biomarkers that may be used in the early detection of lymph node metastasis in these patients. A total of 227 different metabolites were identified in the serum of patients with stage T3 colon cancer with or without lymph node metastasis, of which, 17 were able to distinguish patients with lymph node metastasis from those without. Five factors, including the presence of abscisic acid, calcitroic acid and glucosylsphingosine in the serum, age and sex were independent predictors for lymph node metastasis, and the three metabolites abscisic acid, calcitroic acid and glucosylsphingosine were independent predictors for the survival of patients. Increased serum levels of abscisic acid, calcitroic-acid and glucosylsphingosine may therefore be considered as potential biomarkers to predict lymph node metastasis and survival in patients with colon cancer. The results from the present study provided crucial information regarding the use of serum metabolites as biomarkers for patients with colon cancer; however, further investigation is required to validate the present data before clinically applying this method.

Previous studies have identified different serum metabolites biomarkers of colon cancer or predictive markers for colon cancer prognosis (18–22,28). For example, a previous study demonstrated that the Fourier transform ion cyclotron resonance mass spectrometry can determine serum metabolites for the early detection and screening of colorectal cancer (18). Furthermore, a recent study identified 404 serum metabolites, of which 50 were differentially represented between patients with colorectal cancer or colorectal adenoma polyps and healthy subjects (19). Another study used NMR and LC-MS spectra for the determination of serum metabolites, in order to distinguish patients with normal colorectum from those with colorectal adenoma polyps or colorectal cancer (20). A previous study reported that five metabolites, including succinate, N2,N2-dimethylguanosine, adenine, citraconic acid and 1-methylguanosine, can be used to detect colorectal cancer with a sensitivity of 0.83, specificity of 0.94 and area under the receiver operator characteristic curve (AUROC) of 0.91. Conversely, the values of sensitivity, specificity and AUROC for carcinoembryonic antigen as a biomarker of colorectal cancer are 0.75, 0.76 and 0.80, respectively (21,22). Metabolomics research is feasible and has great potential in the diagnosis of colon cancer, for example a previous study screened a group of urinary metabolites as biomarkers for the early detection of colorectal cancer (18–22,28). The group compared the expression levels of metabolite markers of patients with CRC, including citrate, hippurate, p-cresol, 2-aminobutyrate, myristate, putrescine and kynurenate, with healthy individuals. However, all these studies used a small number of patients (18–22). The present study included a unique cohort of patients with stage T3 colon cancer with and without lymph node metastasis (there were no patients with T3NxM0 colon cancer included). In this regard, the present data are novel and provide useful information. Similarly, a previous study assessed the use of serum metabolites for colorectal cancer staging; however, this study contained only 16 cases (29). A previous study involving 14 patients with stages I–V colorectal cancer identified 139 known metabolites, of which 16 can predict colorectal cancer staging (24). However, the previous and present studies identified very few overlapped metabolites, which may be due to the different populations, the diet and lifestyle of patients, and the methodologies used. The definitive establishment of serum metabolites as biomarkers in colon cancer staging and prognosis is therefore challenging. In addition, a previous study reported that ultra-performance liquid chromatography and quadrupole time-of-fight mass spectrometry with positive electrospray ionization analysis can identify 18 biomarkers with the potential to diagnose ovarian cancer. The metabolites were potential biomarkers to diagnose ovarian cancer, of which 12 were confirmed in the validation cohort of patients (30).

Analysis of the metabolic profile in patients with colon cancer can be used to investigate the underlying metabolic mechanisms of colon cancer, help clinicians better understand the role of different metabolites in carcinogenesis and the progression of colon cancer, and discover candidate biomarkers for the early detection of tumors or metastasis and of treatment responses (31–36). In the present study, 17 serum metabolites were found to be significantly different between patients with colon cancer and lymph node metastasis and patients without. The analysis of the clinicopathological characteristics of these patients, including sex, age, p53 expression, Ki-67 serum level, tumor vessel infiltration, alcohol consumption, smoking history and family history of cancer, demonstrated that sex and age were associated with colon cancer lymph node metastasis. However, these two factors failed to independently predict patient survival, and further investigation is required in order to confirm these findings.

The metabolites identified in the present study included vitamin D metabolic end product calcitroic acid, further confirming the protective role of vitamin D in colon cancer (37,38). Although the underlying mechanism of vitamin D in the prevention of colon cancer remains unclear, some possible mechanisms include inhibition of cell proliferation and stimulation of cell differentiation and apoptosis by vitamin D, which can subsequently inhibit colon carcinogenesis (37,38). A previous study reported that vitamin D and its derivatives can induce the expression of bone morphogenetic protein (BMP) and activate the BMP-Smad signaling pathway (39). The BMP-Smad signaling pathway is involved in the pathway for enzyme-coupled receptor signal transduction, and BMP is one β-tumor necrosis factor (38,40) that regulates cell proliferation, differentiation and apoptosis. Furthermore, previous *in vitro* experiments demonstrated that vitamin D and its metabolites or analogs induce BMP overexpression and activates BMP-Smad signaling pathway to suppress the development of colon cancer (41,42).

The present study presented some limitations. First, the cohort of patients lacked those with early stage colon cancer, distant tumor metastases and the serum was not compared with the postoperative serum. Secondly, the use of Matrix Assisted Laser Desorption Ionisation-Time of Flight (TOF)/TOF-MS may be more appropriate to precisely analyse the two-dimensional difference gel electrophoresis dissected protein spots; however, since this material was not available at the The First Hospital of Jilin University, the present study used LC-MS to analyze the serum samples. Thirdly, some *in vitro* studies are required to further elucidate the pathophysiology of colon cancer.

In conclusion, LC-MS possesses a high potential to analyze clinical samples and investigate the changes in serum metabolites of patients. The present study identified three metabolites, including abscisic acid, calcitroic acid and glucosylsphingosine, as independent risk factors for lymph node metastasis and prognosis in patients with colon cancer. Further investigation using a larger number of sample from numerous institutions is required to validate these finding.

## Figures and Tables

**Table I.** Differential serum metabolites from patients with stage T3 colon cancer and different lymph node status.

No.	Name	Molecular weight, g/mol	Rain time, min	Response, T3Nx	Response, T3N0	P-value
M1	Tyramine	137.179	10.56	NA	193.34±415.58	8.90×10^−4^
M2	Abscisic acid	264.3169	9.81	1.51±6.79	18.2±38.94	2.40×10^−3^
M3	3-hydroxynonanoyl carnitine	317.421	9.93	3.88±14.55	21.13±58.78	1.90×10^−3^
M4	Ethanolamine Oleate	61.0831	7.29	5.63±12.37	24.99±30.27	3.14×10^−5^
M5	Coutaric acid	349.4247	9.93	0.65±2.77	13.91±33.18	4.20×10^−3^
M6	Sorgoleone	358.4712	9.87	16.32±28.83	1.71±5.65	4.00×10^−4^
M7	Aldosterone	360.444	7.32	56.22±66.11	5.35±16.2	2.75×10^−7^
M8	Calcitroic acid	374.5137	9.88	6.49±17.48	44.19±80.73	1.10×10^−3^
M9	Lithocholic acid	376.5726	9.89	3.92±14.50	35.54±68.93	1.32×10^−3^
M10	Cinncassiol C3	382.448	7.10	114.23±125.3	31.66±65.46	3.92×10^−5^
M11	Treprostinil	390.5131	9.44	0.89±4.93	33.61±75.71	2.00×10^−3^
M12	Flavoxate	391.4596	5.24	2.63±6.13	14.33±34.15	1.40×10^−2^
M13	Hydroxy-5-(3′,5′-dihydroxyphenyl)-valeric acid-O-glucuronide	402.35	5.68	399.23±697.43	79.95±259.43	2.10×10^−3^
M14	Phenobarbital O-glucuronide	232.2353	5.69	148.41±291.76	17.46±68.4	1.70×10^−3^
M15	Pinostrobin 5-glucoside	432.4206	5.85	43.13±100.18	5.8±16.01	7.90×10^−3^
M16	Lithocholic acid glycine conjugate	433.6239	9.91	2.66±9.79	24.6±49.85	1.90×10^−3^
M17	Glucosylsphingosine	461.6325	6.74	40.9±59.64	95.84±77.24	7.07×10^−5^

The data are expressed as the mean ± standard deviation and statistically analyzed using the two-sample t-test. Rain time is the time that the metabolites are detected. Response is the average of the values we detected in all samples of the group. NA, not available.

**Table II.** Univariate logistic regression analysis of differential serum metabolites and clinical features for association with lymph node metastasis.

						95% CI	
						
Variables	B	SE	Wald	P-value^a^	OR	Lower	Upper
Abscisic acid	−0.044	0.017	6.891	0.009	0.957	0.92	0.98
Calcitroic acid	0.018	0.006	8.569	0.003	1.018	1.00	1.03
Glucosylsphingosine	−0.012	0.004	6.778	0.009	0.988	0.98	0.99
Ethanolamine Oleate	−0.019	0.009	3.909	0.048	0.982	0.964	1.000
Coutaric acid	−0.008	0.008	0.838	0.360	0.992	0.977	1.009
Aldosterone	0.005	0.002	5.497	0.019	1.005	1.001	1.008
Lithocholic acid	0.001	0.002	0.357	0.550	1.001	0.997	1.006
Cinncassiol C3	0.003	0.002	3.565	0.059	1.003	1.000	1.007
Treprostinil	0.001	0.002	0.083	0.773	1.001	0.996	1.005
Flavoxate	0.002	0.004	0.254	0.614	1.002	0.994	1.010
Age	0.072	0.024	9.371	0.002	1.075	1.026	1.126
Sex	−1.141	0.600	3.829	0.050	0.320	0.101	1.007
Tumor size	−0.113	0.101	1.256	0.262	0.893	0.732	1.089
P53	0.000	0.006	0.002	0.964	1.000	0.988	1.011
Ki-67	0.007	0.017	0.163	0.686	1.007	0.974	1.041
Vascular infiltration	0.323	0.457	0.499	0.480	1.381	0.564	3.385
Alcohol consumption	−0.214	0.620	0.119	0.731	0.808	0.240	2.723
Smoking history	−0.054	0.473	0.013	0.909	0.947	0.375	2.394
Family tumor history	−0.643	1.119	0.330	0.566	0.526	0.059	4.710

a P-values were generated using χ^2^ test. CI, confidence interval; SE, standard error; B, regression coefficients; Wald, Wald test result; OR, odds ratio.

**Table III.** Multivariate Cox regression analysis of differential serum metabolites and clinical features for association with lymph node metastasis.

						95% CI	
						
Variables	B	SE	Wald	P-value^a^	OR	Lower	Upper
Abscisic acid	−0.044	0.017	6.891	0.009	0.957	0.92	0.98
Calcitroic acid	0.018	0.006	8.569	0.003	1.018	1.00	1.03
Glucosylsphingosine	−0.012	0.004	6.778	0.009	0.988	0.98	0.99
Age	0.090	0.028	10.444	0.001	1.094	1.03	1.15
Sex	−1.141	0.586	3.793	0.051	0.320	0.10	1.00

a P-values were generated using χ^2^ test. CI, confidence interval; SE, standard error; B, regression coefficients; Wald, Wald test result; OR, odds ratio.

## Data Availability

The datasets used and/or analyzed during the current study are available from the corresponding author on reasonable request.
